# Cellular Entry of Ebola Virus Involves Uptake by a Macropinocytosis-Like Mechanism and Subsequent Trafficking through Early and Late Endosomes

**DOI:** 10.1371/journal.ppat.1001110

**Published:** 2010-09-16

**Authors:** Mohammad F. Saeed, Andrey A. Kolokoltsov, Thomas Albrecht, Robert A. Davey

**Affiliations:** 1 Department of Microbiology & Immunology, The University of Texas Medical Branch, Galveston, Texas, United States of America; 2 Galveston National Laboratory, The University of Texas Medical Branch, Galveston, Texas, United States of America; 3 Institute of Human Infection and Immunity, The University of Texas Medical Branch, Galveston, Texas, United States of America; 4 Department SK, Building 37, NASA, Houston, Texas, United States of America; Mount Sinai School of Medicine, United States of America

## Abstract

*Zaire ebolavirus* (ZEBOV), a highly pathogenic zoonotic virus, poses serious public health, ecological and potential bioterrorism threats. Currently no specific therapy or vaccine is available. Virus entry is an attractive target for therapeutic intervention. However, current knowledge of the ZEBOV entry mechanism is limited. While it is known that ZEBOV enters cells through endocytosis, which of the cellular endocytic mechanisms used remains unclear. Previous studies have produced differing outcomes, indicating potential involvement of multiple routes but many of these studies were performed using noninfectious surrogate systems such as pseudotyped retroviral particles, which may not accurately recapitulate the entry characteristics of the morphologically distinct wild type virus. Here we used replication-competent infectious ZEBOV as well as morphologically similar virus-like particles in specific infection and entry assays to demonstrate that in HEK293T and Vero cells internalization of ZEBOV is independent of clathrin, caveolae, and dynamin. Instead the uptake mechanism has features of macropinocytosis. The binding of virus to cells appears to directly stimulate fluid phase uptake as well as localized actin polymerization. Inhibition of key regulators of macropinocytosis including Pak1 and CtBP/BARS as well as treatment with the drug EIPA, which affects macropinosome formation, resulted in significant reduction in ZEBOV entry and infection. It is also shown that following internalization, the virus enters the endolysosomal pathway and is trafficked through early and late endosomes, but the exact site of membrane fusion and nucleocapsid penetration in the cytoplasm remains unclear. This study identifies the route for ZEBOV entry and identifies the key cellular factors required for the uptake of this filamentous virus. The findings greatly expand our understanding of the ZEBOV entry mechanism that can be applied to development of new therapeutics as well as provide potential insight into the trafficking and entry mechanism of other filoviruses.

## Author Summary

Filoviruses, including *Zaire ebolavirus* (ZEBOV), are among the most pathogenic viruses known. Our understanding of how these viruses enter into host cells is very limited. A deeper understanding of this process would enable the design of better targeted antiviral therapies. This study defines in detail, key steps of ZEBOV cellular uptake and trafficking into cells using wild type virus as well as the host factors that are responsible for permitting virus entry into cells. Our data indicated that the primary mechanism of ZEBOV uptake is a macropinocytosis-like process that delivers the virus to early endosomes and subsequently to late endosomes. These findings aid in our understanding of how filoviruses infect cells and suggest that disruption of macropinocytosis may be useful in treatment of infection.

## Introduction

*Zaire ebolavirus* (ZEBOV, Genbank:AF086833), a member of the family *Filoviridae*, genus *Filovirus*, causes a highly fatal hemorrhagic fever in humans and non-human primates. Over the past three decades numerous human outbreaks have occurred in Central Africa involving hundreds of cases with fatality rates ranging from 50–89% [1]. In addition, outbreaks of ZEBOV infection have been implicated in deaths of tens of thousands of gorillas, chimpanzees and duikers in Central and Western Africa posing a considerable threat to the wildlife and ecology in those areas [2]. Due to a very high case fatality rate in humans, significant transmissibility of the virus, lack of effective preventive or therapeutic measures against the disease, ZEBOV is considered a serious emerging viral pathogen. Currently no specific therapy or vaccine is approved for human or animal use against this pathogen.

As for other members of *Filoviridae*, ZEBOV is morphologically distinct from other animal viruses. The virions are long and filamentous with an average length of 800–1000 nm and a diameter of about 80 nm but can form a variety of shapes ranging from straight rods to closed circles [3]. Virions are surrounded by a host cell-derived lipid envelope. The envelope contains virally-encoded glycoprotein (GP) spikes composed of homotrimers of two virally-encoded glycoproteins, GP1 and GP2. The approximately 19 kb single-stranded, negative-sense genomic RNA complexed with nucleocapsid, VP35, VP30 and L proteins form the nucleocapsid, while VP40 forms the matrix that underlies the viral membrane [4].

Like all viruses, ZEBOV largely relies on host cell factors and physiological processes for key steps of its replication cycle. Identification of these processes and factors will not only allow a better insight into pathogenic mechanism, but may identify novel targets for future therapeutic development. As the first step of replication, entry into the host cell is an attractive target for therapeutic intervention as infection can be stopped before virus replication disrupts cellular functions. However, the entry mechanism of ZEBOV, and that of other large enveloped viruses is very limited.

Many enveloped viruses, including ZEBOV, require endocytosis to infect cells. The internalized virus is transported through successive endocytic vesicles to reach a vesicle/compartment where conditions are conducive (low pH and/or presence of proteolytic enzymes) for the GP to attain a suitable conformation needed for membrane fusion [5], [6], [7]. Upon fusion of viral and endocytic membranes, the capsid moves into the cell cytoplasm to begin genome replication. Several distinct endocytic mechanisms exist in mammalian cells. They are distinguished from each other on a number of criteria including the size and morphology of endocytic vesicles, the type of cargo they carry, the cellular factors involved in their control and their origins and destinations [8]. Different viruses employ different routes of endocytosis, and the route taken by a given virus largely depends on the receptor it interacts with.

Clathrin-mediated endocytosis (CME) is the best understood endocytic pathway. A number of viruses including Influenza A (Genbank:M73524), Semliki forest (Genbank:X04129) and vesicular stomatitis viruses (VSV; Genbank:J02428) employ this pathway for entry [9], [10]. The internalization of the virus-receptor cargo occurs in specialized areas of cell membrane called clathrin-coated pits (CCPs). CCPs are formed on the cytoplasmic face of the plasma membrane through sequential assembly of proteins including clathrin that form a cage-like structure lining the cytoplasmic side of the pit. The pit then invaginates and buds from the plasma membrane forming a clathrin-coated vesicle approximately 120 nm in diameter containing the internalized cargo. Subsequently, the vesicle sheds its clathrin coat, a prerequisite for further trafficking and merging with other compartments. A number of accessory, adaptor and signaling molecules participate in this process, and provide a tight regulation of the pathway. Some, such as accessory protein-2 (AP2) and Eps15, are specifically associated with CCPs, while others such as dynamin, which is responsible for vesicle budding from the plasma membrane, are shared with other endocytic pathways [8].

Caveolin-mediated endocytosis (CavME), first observed for the cellular uptake of simian virus 40 (SV40) [11], differs from CME in terms of internalization mechanism and vesicular transport route. Caveolae are flask-shaped invaginations in the plasma membrane that are rich in caveolin protein, and are predominantly associated with cholesterol-rich plasma membrane microdomains termed lipid rafts. Therefore, extraction or perturbation of membrane cholesterol severely impedes entry of viruses that use CavME. Vesicles derived from CavME are indicated by the presence of caveolin and are termed caveosomes. Other cellular factors such as Eps15-related (Eps15R) protein are thought to be specific for CavME, but as with CME, dynamin is still required for severing of caveolae from the plasma membrane. Another distinguishing feature is that caveloae are smaller than CCPs and have an average diameter of approximately 60–80 nm [8].

Recently, the importance of macropinocytosis, as a distinct endocytic uptake mechanism for virus infection, has started to be realized for some viruses [9]. Macropinocytosis is associated with membrane ruffles such as those formed by filopodia and lamellipodia, which are outward extensions of the plasma membrane driven by actin polymerization underneath the membrane surface [12]. When a ruffle folds back upon itself a cavity can be formed. Subsequent fusion of the distal end of the loop with the plasma membrane results in formation of a large vesicle called a macropinosome. These can range in size from 200 to 10,000 nm across and take up cargoes of similar dimensions [9]. Morphological and regulatory characteristics that distinguish macropinocytosis from other endocytic processes have also begun to emerge [8], [9], [13], [14]. Macropinosomes are best characterized for uptake of fluid phase markers such as high molecular weight dextran and horse-radish peroxidase and is sensitive to inhibitors of Na+/H+ exchangers, such as amilorides [14]. As for CavME, they are dependent on cholesterol-rich lipid rafts, but dynamin is not required. Instead, scission of macropinosomes appears to require CtBP/BARS [9], [15], [16]. Other work indicates the involvement of cell signaling factors PI3K, Akt, PKC, PLCγ and PLC-A_2_ that act to promote membrane ruffling by stimulating actin remodeling through Rac and cdc42 [9].

All endocytic pathways used by viruses serve to deliver virus to vesicles and compartments conducive to virus membrane fusion and release of the core into the cell cytoplasm at a site where replication proceeds optimally. Many endocytic pathways share common features, such as acidification, yet each virus type appears to prefer one trafficking pathway over others and misdirection into alternative pathways can result in inhibition of infection. For most enveloped viruses, the point at which membrane fusion occurs appears to be at the early or late endosome stage. This evidence has been gathered by comparing pH-sensitivity of the GP to known pH of the endosome at different stages of maturation. More recently, the use of dominant negative GTPases, that are involved in endosomal maturation, have been used in determining virus exit points from the endosome [17], [18], [19]. In general, the two methods agree but provide little detail as to whether viruses have additional requirements in terms of site of release other than the early or late endosome.

Currently, a detailed understanding of ZEBOV endocytosis and trafficking is lacking. Each of the previous studies on understanding ZEBOV entry pathway have indicated involvement of different pathways, including CME [20], [21], CavME [20], [22] and a Rho GTPase-dependent pathway that may suggest involvement of macropinocytosis [23]. These conflicting findings may be due to the use of surrogate models of ZEBOV such as pseudotyped retroviruses, which are morphologically and biochemically distinct from wild type filamentous ZEBOV and/or reliance on one analytical approach, such as use of pharmacological agents, which are likely to act on more than one cellular target [24].

Here we have used multiple independent approaches employing replication-competent, infectious ZEBOV and/or morphologically comparable virus-like particles (VLPs). We have examined the contribution of each endocytic pathway to ZEBOV entry and infection by quantitative analysis. The work involves measuring the impact of drugs, siRNA and/or expression of well characterized dominant negative (DN) mutants of cell trafficking proteins on virus entry and infection. We also use fluorescently-labeled virus-like particles (VLPs) to follow virus internalization and trafficking through different endocytic compartments. The product of combining all these approaches provides, for the first time, an accurate and detailed description of ZEBOV uptake mechanism. Our data clearly indicate that wild type ZEBOV enters and infects Vero and HEK293T cells independently of clathrin, caveolae and dynamin. Instead, virus entry required the presence of cholesterol in the cell membrane and was inhibited by the amiloride derivative, EIPA. A marked induction in fluid-phase uptake was also observed shortly after virus binding to cells and internalized virus particles showed significant colocalization with high molecular weight dextran. In addition, inhibition of p53-activated kinase (Pak1) or CtBP/BARS resulted in significant reduction in virus entry and infection. Importantly, the virus particles appear to stimulate uptake through this pathway directly by promoting localized actin polymerization and this is consistent with our previous work where the GP triggered the PI3 kinase signaling cascade and Rac1 activity [25]. No evidence for involvement of clathrin- or caveolae-dependent endocytosis was seen. Instead, the primary mechanism of virus uptake appears closely related to macropinocytosis. Subsequent to internalization, the virus utilizes the conventional endolysosomal pathway and is trafficked through early and late endosomes before membrane fusion takes place. This study provides novel information regarding ZEBOV entry, and is likely to be useful in understanding the entry mechanism of other filoviruses.

## Results

### Clathrin and caveolar endocytosis are not involved in ZEBOV virus entry

**Figure 1:**
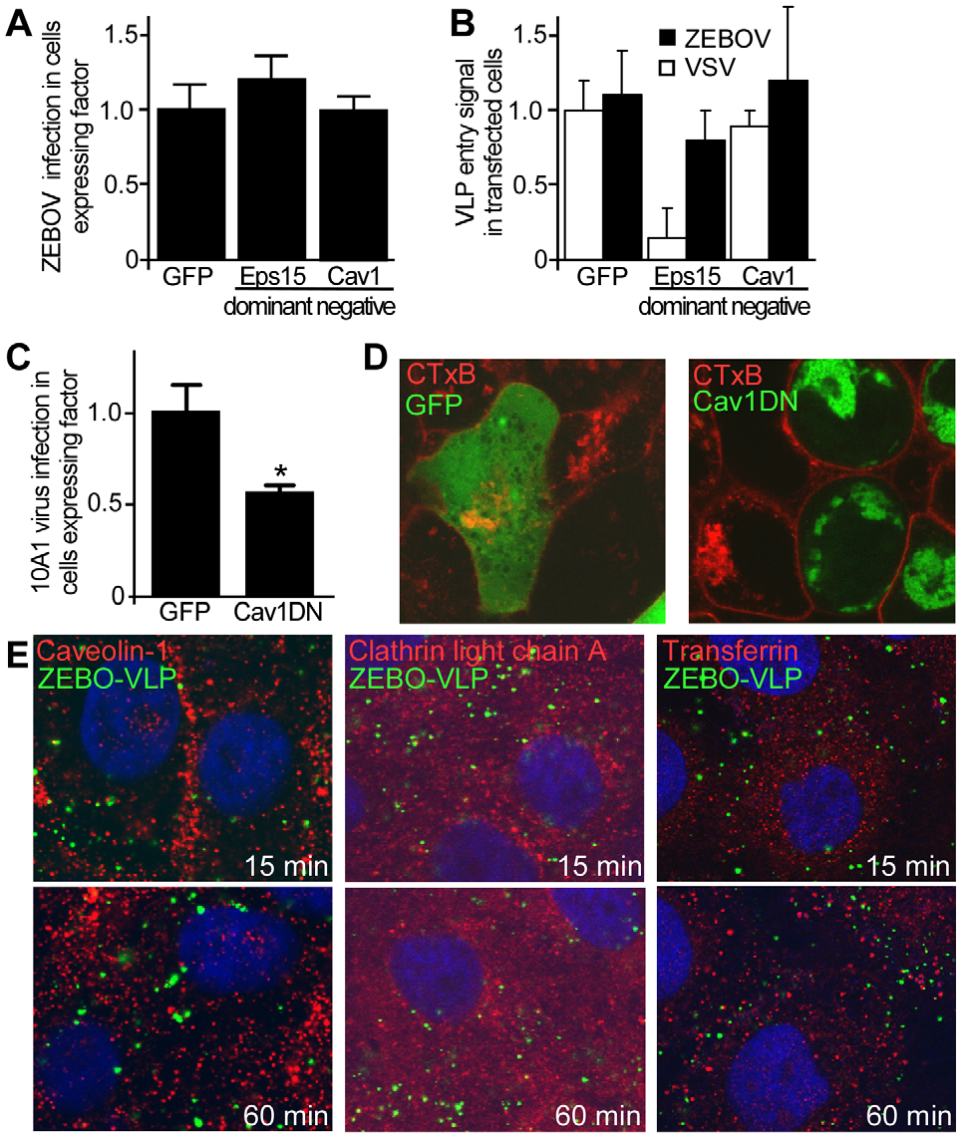
Clathrin and caveolar endocytosis are not required for ZEBOV entry. (**A**) **Regulators of clathrin and caveolae-mediated endocytosis are not important for ZEBOV infection.** The role of proteins important for endocytosis in ZEBOV infection was assessed using dominant negative (DN) effector proteins. HEK293T cells were transfected with plasmids encoding GFP, DN-Eps15-GFP or DN-Cav1-GFP. Twenty-four h post-transfection cells were inoculated with wild-type ZEBOV (MOI  = 0.2). Cells were fixed 48 h later, stained for nuclei using DAPI and for ZEBOV VP40 matrix protein using a specific rabbit antiserum followed by Alexa_633_ secondary antibody. Images were taken by fluorescence microscopy and analyzed as described in the methods. To quantitate the infection dependency of ZEBOV on expression of each construct, the proportion of cells that were expressing each GFP-tagged fusion protein and infected by ZEBOV was calculated as a fraction of the total cell population. The data were averaged for all replicates (>5) and normalized to that seen in cells transfected with GFP alone. (**B**) To measure the impact of expression of each GFP-tagged protein on the virus entry step into cells, a contents mixing assay was performed. Cells were transfected as above and then used in the assay 36 h after transfection. Both ZEBO-VLPs and VSV-G pseudotyped particles were used as indicated. Measurements were made at 3 h, at which time the contents mixing signal peaked in untreated cells (peak is at 2–3 h post cell binding). Measurements were normalized to untransfected cells. The results are mean ± st.dev. of 3 independent experiments. (**C**) To test DN-Cav1 efficacy, HEK293 cells were transfected with plasmids encoding GFP or DN-Cav1 tagged with GFP. Thirty six hours after transfection cells were infected with a recombinant 10A1 MLV virus encoding a truncated CD4 receptor as a marker for infection. 36 h after the infection cells were stained for CD4 expression with anti-CD4 antibody conjugated to PE (red) and cells expressing CD4 and the GFP-tagged protein by microscopy. Data were analyzed as described in the methods and in (A). (**D**) **Cholera toxin B subunit uptake is blocked in cells expressing DN-Cav-1.** As an additional test of DN-Cav-1 efficacy, the impact of expression on cholera toxin subunit B (CTxB) uptake was measured. HEK293T cells were transfected with plasmid encoding GFP (left panel) or GFP-tagged DN-Cav1 protein (right panel). Thirty-six h after transfection cells were incubated with fluorescently-labeled CTxB for 30 or 60 min, fixed and imaged. Images were taken by confocal microscopy with a mid z-section shown. Green  =  GFP or DN-Cav1; Red  =  CTxB. (**E**) **ZEBO-VLPs do not associate with markers of caveolae or clathrin-coated endosomes.** Vero cells were preincubated with gfpZEBO-VLPs at 16°C (to prevent endocytosis) for 15 min to allow virus attachment. Excess virus was then removed and the temperature raised to 37°C (to initiate endocytosis) prior to fixation at indicated times. For caveolin-1 and clathrin light chain A, permeabilized cells were stained with anti-Cav1 antibody or anti-CLCA antibody followed by Alexafluor_594_-conjugated secondary antibody. For transferrin, Alexafluor_594_-labeled transferrin was added to cells during incubation with the VLPs. DAPI was used to stain nuclei (blue). Images were taken by confocal microscopy with a mid z-section shown. Green  =  gfpZEBO-VLPs; Red  =  indicated endocytic marker.

A recent study suggested that Ebola virus uses CME for cellular entry [21], while an earlier study had implicated both CME and CavME [20], [21]. However, these studies utilized either pseudotyped virus, which due to morphological and/or biochemical differences may not accurately depict the ZEBOV entry pathway, and/or relied solely on the use of pharmacological agents, which may alter multiple processes important for membrane trafficking. To more closely examine the role of the each endocytic pathway we used replication-competent infectious virus and morphologically comparable ZEBOV virus-like particles (VLPs) to determine the impact of specific dominant-negative (DN) forms of Eps15 (OMIM:600051) and caveolin-1 (cav-1, OMIM:601047) on infection and VLP uptake into cells. Eps15 and caveolin-1 (cav-1) are required for the formation and trafficking of CME and CavME vesicles respectively, and their DN forms inhibit the respective endocytosis with high specificity [26]. HEK293T cells were transfected with plasmid encoding GFP alone or GFP-tagged forms of DN-Eps15 or DN-Cav1, and subsequently infected with ZEBOV. Infected cells were detected by immunofluorescence staining for ZEBOV matrix protein (VP40) protein and the proportion of cells that co-expressed the transfected protein and ZEBOV VP40 (infection marker) was calculated. It was found that the proportion of ZEBOV-infected cells in cultures expressing DN-Eps15-GFP or DN-Cav1-GFP was not significantly different (P>0.05) to that in cultures expressing GFP alone, indicating that neither DN protein had a significant impact on ZEBOV infection (Fig. 1A). This lack of effect of either DN protein on ZEBOV entry was confirmed using a sensitive contents-mixing entry assay that measures virus-endosomal membrane fusion by monitoring luciferase release from VLPs into the cell cytoplasm [25]. Expression of either DN protein failed to have any significant effect on entry of ZEBO-VLP (P>0.05; Fig. 1B). As control, VLPs bearing VSV envelope glycoprotein (VSV-VLP) were used. VSV is known to use CME for cellular entry [27]. Entry of VSV-VLP was significantly inhibited only in cells expressing DN-Eps15 (Fig.1B, P<0.001). To confirm that DN-Cav1 expression impacted caveolar endocytosis, murine leukemia virus 10A1 (MLV-10A1) infection and cholera toxin B subunit (CTxB, Pubchem:53787834) uptake were measured as both processes are known to require CavME [28], [29]. 10A1 infection of cells expressing DN-Cav-1 was reduced by half (Fig. 1C) and is consistent with previously reported observations [28]. Uptake of CTxB (Fig. 1D) was more strongly inhibited, as cells expressing DN-Cav1 had little CTxB inside the cytoplasm, indicating that DN-Cav1 was functional, blocking caveolar endocytosis.

As a further test, colocalization of internalized GFP-labeled ZEBO-VLPs (gfpZEBO-VLP) with established markers of CME (clathrin light chain A; OMIM:118960 and transferrin; OMIM:190000) or CavME (caveolin-1) pathways was examined. Confocal microscopy revealed no significant colocalization of gfpZEBO-VLP with any of the markers used (Fig. 1E). Similar results were obtained when Vero cells were used (not shown). Taken together, the above findings indicated that neither CME nor CavME plays a major role in entry and infection of ZEBOV into HEK293T or Vero cells.

### Dynamin is not required for ZEBOV entry

**Figure 2:**
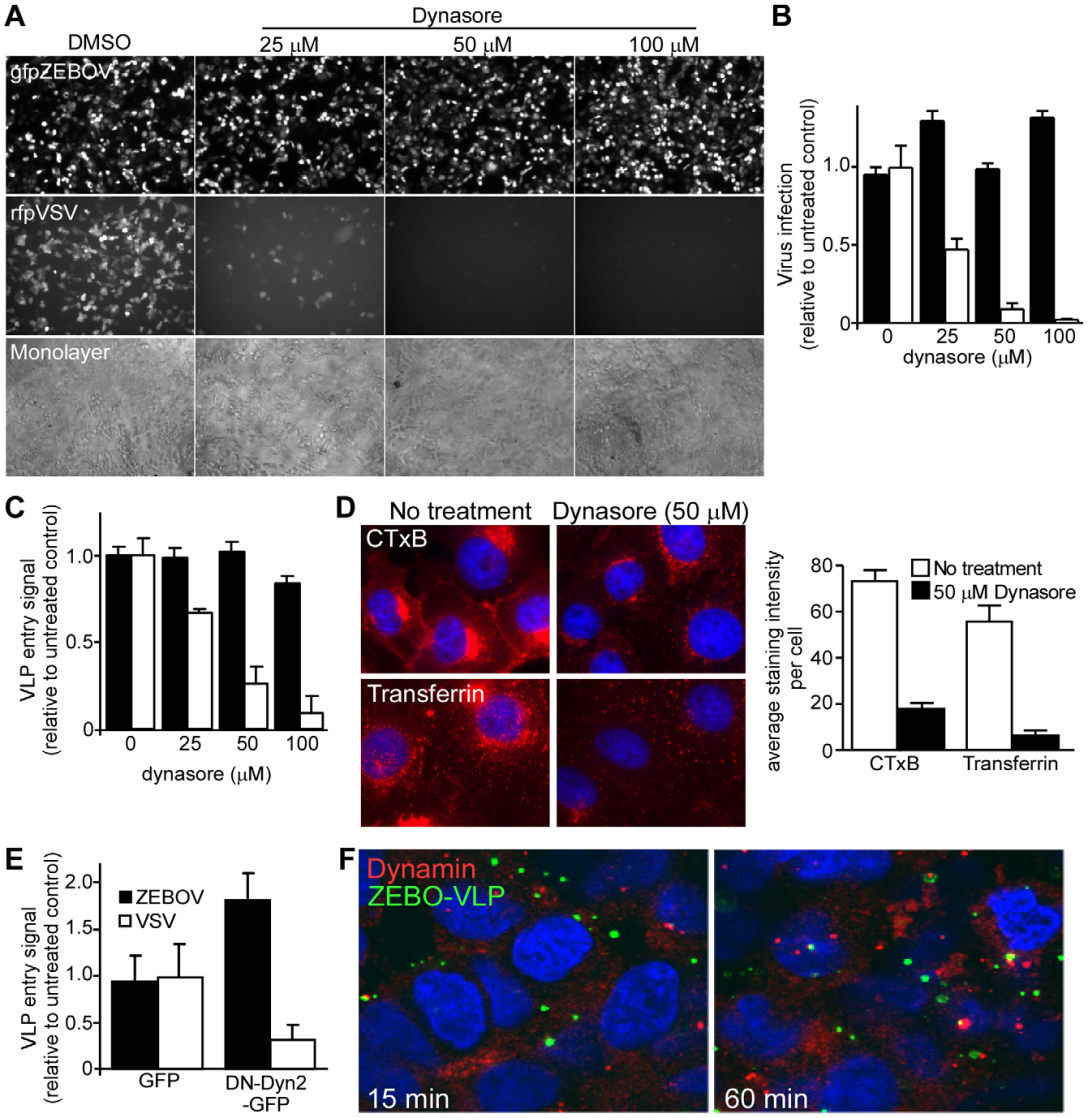
ZEBOV entry does not require dynamin activity. (**A**) **Dynasore does not affect ZEBOV infection.** Vero cells pretreated with the indicated doses of dynasore were incubated with either GFP-expressing infectious ZEBOV (gfpZEBOV, top panel) or RFP-expressing infectious VSV (rfpVSV, middle panel) in the continued presence of the drug (MOI  = 0.1). After 24 h, cells were washed and fixed with 10% formalin and images taken using an epifluorescence microscope. Phase-contrast microscopy was also performed to ensure cell monolayers were intact (bottom panel). (**B**) The bar graph shows quantitation of data shown in (A). For this, green fluorescent cells were counted using Cell Profiler software (Broad Inst., MA) and normalized to the average of the untreated control. At least 4 sets of images were analyzed. Solid bars represent infection by gfpZEBOV and open bars represent rfpVSV. Similar results were obtained with HEK293T cells (not shown). (**C**) **Dynasore does not affect cell entry of ZEBO-VLPs.** Entry assays were performed with HEK293T cells after pre-incubation with dynasore for 1 h. Cells were then challenged with luciferase containing ZEBO-VLP (solid bars) or VSV-VLP (open bars) for 3 h in the continued presence of the drug. Subsequently, cells were washed and incubated with luciferase assay buffer and luciferase activity was measured. The results are expressed as luciferase activity relative to that in untreated cells. The data represents average ± st.dev. of 3 independent experiments each performed in duplicate. (**D**) **Dynasore blocks CTxB and transferrin uptake.** To confirm the activity of dynasore, Vero cells (untreated or pre-treated with 50 µM dynasore) were incubated with Alexafluor_594_-labeled cholera toxin B subunit (CTxB) or transferrin (both red). After 1 h, cells were fixed and analyzed by fluorescence microscopy for uptake of each marker as indicated. Nuclei (blue) were stained with DAPI. The bar graph (right panel) shows the relative amount of each probe taken up by cells, which was determined by calculating the mean pixel intensity of the probe signal per unit area and expressed as the average of 10 cells. (**E**) **Dominant negative dynamin does not affect ZEBO-VLP cell entry.** Effect of DN-dynamin on VLP entry was determined by using HEK293T cells transfected with plasmid encoding GFP alone or DN dynamin2 (K44A)-GFP fusion protein (DN-Dyn2-GFP). Twenty-four h post-transfection, cells were incubated with ZEBO-VLP or VSV-VLP for 3 h. Luciferase activity was then measured in each sample and expressed relative to that in control (untransfected) cells. The data represents average ± st.dev. of 3 independent experiments, each performed in duplicate. (**F**) **ZEBO-VLPs do not colocalize with endogenous dynamin.** VLP colocalization with dynamin was tested by binding gfpZEBO-VLPs to HEK293T cells at 16°C for 15 min. Cells were then shifted to 37°C to allow VLP uptake and fixed at 15 or 60 min. They were then permeabilized and stained with anti-dynamin-2 antibody and Alexafluor_594_-conjugated secondary antibody. Images were taken with a confocal microscope with mid z-sections shown. Nuclei (blue) were stained with DAPI. Green  =  gfpZEBO-VLPs; Red  =  endogenous dynamin-2.

Dynamin (OMIM:602377) is a large GTPase and plays a critical role in numerous endocytic pathways including CME and CavME as well as some of the non-clathrin/non-caveolin-dependent (NC) pathways [8]. Dynamin acts by mediating the release of newly-formed endocytic vesicles from the plasma membrane. To determine ZEBOV dependence on dynamin, the effect of dynasore (Pubchem:56437635), a potent and specific dynamin inhibitor [30] was tested. A recombinant infectious ZEBOV that encodes GFP (gfpZEBOV) was used. This virus is comparable to wild-type ZEBOV in terms of replication and cytopathic effects (CPE) in cultured cells but has been engineered to express GFP as an infection marker [31]. As control, a recombinant infectious VSV that encoded red fluorescent protein (rfpVSV) was used. Dynasore treatment of Vero cells greatly reduced rfpVSV infection but failed to have any significant effect on infection by gfpZEBOV even at the highest concentration tested (Fig. 2A, B). Similar results were obtained in HEK293T cells (not shown). This result was confirmed using the VLP-based entry assay. Just like the gfpZEBOV, entry of ZEBO-VLP was unaffected at any of the doses used while VSV-VLPs were strongly inhibited by dynasore in a dose-dependent manner (Fig. 2C). To ensure that dynasore inhibited dynamin-mediated endocytosis, its effect on internalization of transferrin (CME marker) or CTxB (CavME marker) was determined. Confocal microscopy revealed that treatment reduced internalization of both markers by >80% and 96% respectively in Vero cells (Fig. 2D).

As a further test of the dynamin independence of ZEBOV infection cells were made to express a DN form of dynamin-2 (Dyn2-K44A) and VLP entry assays were performed. As with dynasore, there was a significant drop in the entry of VSV-VLP in cells transfected with Dyn2-K44A (P<0.05). In contrast, the entry of ZEBO-VLPs actually increased significantly (P<0.05), suggesting that the suppression of dynamin function may enhance entry of ZEBOV (Fig. 2E). Furthermore, the majority of GFP labeled ZEBO-VLPs (gfpZEBO-VLPs) did not colocalize with endogenous dynamin at any point up to 60 min after cell contact (Fig. 2F). These findings indicated that cell entry of ZEBOV is independent of dynamin and that dynamin activity may actually redirect virus to a non-productive pathway.

### ZEBOV entry associates with lipid rafts and requires free membrane cholesterol

**Figure 3:**
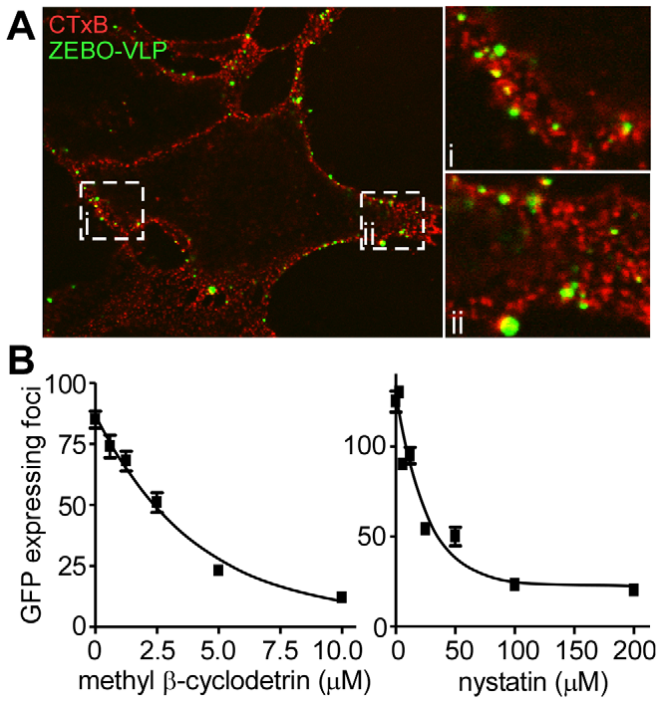
Cholesterol-enriched lipid raft microdomains are important for ZEBOV entry. (**A**) **ZEBO-VLPs associate with lipid rafts.** Vero cells were incubated with gfpZEBO-VLP (green) at 37°C for 15 min and unbound virus was removed by washing. Lipid rafts were visualized by first incubating the cells with Alexafluor_594_-labeled CTxB (red) followed by coalescing the small raft domains with anti-CTxB antibody. The samples were then fixed and images taken by confocal microscopy. A mid z-section of the cells is shown. Insets i and ii are enlarged images of the indicated areas. (**B**) **Cholesterol sequestering drugs inhibit ZEBOV infection.** Vero cells were pretreated with the indicated concentrations of methyl-β cyclodextrin or nystatin for 1 h. Cells were then washed extensively to remove the drugs, and gfpZEBOV was added at an MOI of 0.1. After 24 h, cells were washed and fixed. Images were then taken with a 10× objective lens. The number of foci of infected (gfp-expressing) cells were counted for 4 images per sample in duplicate. The average number of foci is indicated ± st.dev. Similar results were obtained with HEK293T cells (not shown).

Many of the cellular endocytic pathways including CavME, macropinocytosis and certain NC pathways occur in cholesterol-rich membrane microdomains such as lipid rafts. An earlier study suggested that lipid rafts may play a role in ZEBOV infection [32]. Consistent with this, we found significant co-localization of gfpZEBO-VLPs with lipid rafts during entry (Fig. 3A). Furthermore, methyl-β cyclodextrin (Pubchem:3889506) or nystatin (Pubchem:6433272), which disrupt lipid rafts by extracting or sequestering cholesterol out of the plasma membrane, respectively were able to block gfpZEBOV infection in a dose-dependent manner (Fig. 3B). Similar effects of both drugs were observed when tested using the ZEBO-VLP-based entry assay (not shown). These data indicated that cholesterol-rich lipid raft domains are the likely site of ZEBOV entry.

### ZEBOV entry and infection are sensitive to amiloride treatment

The above findings clearly indicated that ZEBOV uptake occurs through a dynamin-independent, lipid raft-dependent, non-clathrin/non-caveolar endocytic mechanism but remains cholesterol dependent. Macropinocytosis is one pathway that is known to be cholesterol-dependent, but independent of clathrin, caveolin and dynamin and has been shown important for uptake of vaccinia virus into cells as well as bacteria [9]. Also, our previous work indicated involvement of PI3K and Rac1 (a rho family GTPase) in ZEBOV entry and infection [25]. Work by others had also indicated involvement of Rho GTPase in Ebola virus entry [23]. Each of these signaling proteins is also thought to be important for macropinocytosis [13], [33].

**Figure 4:**
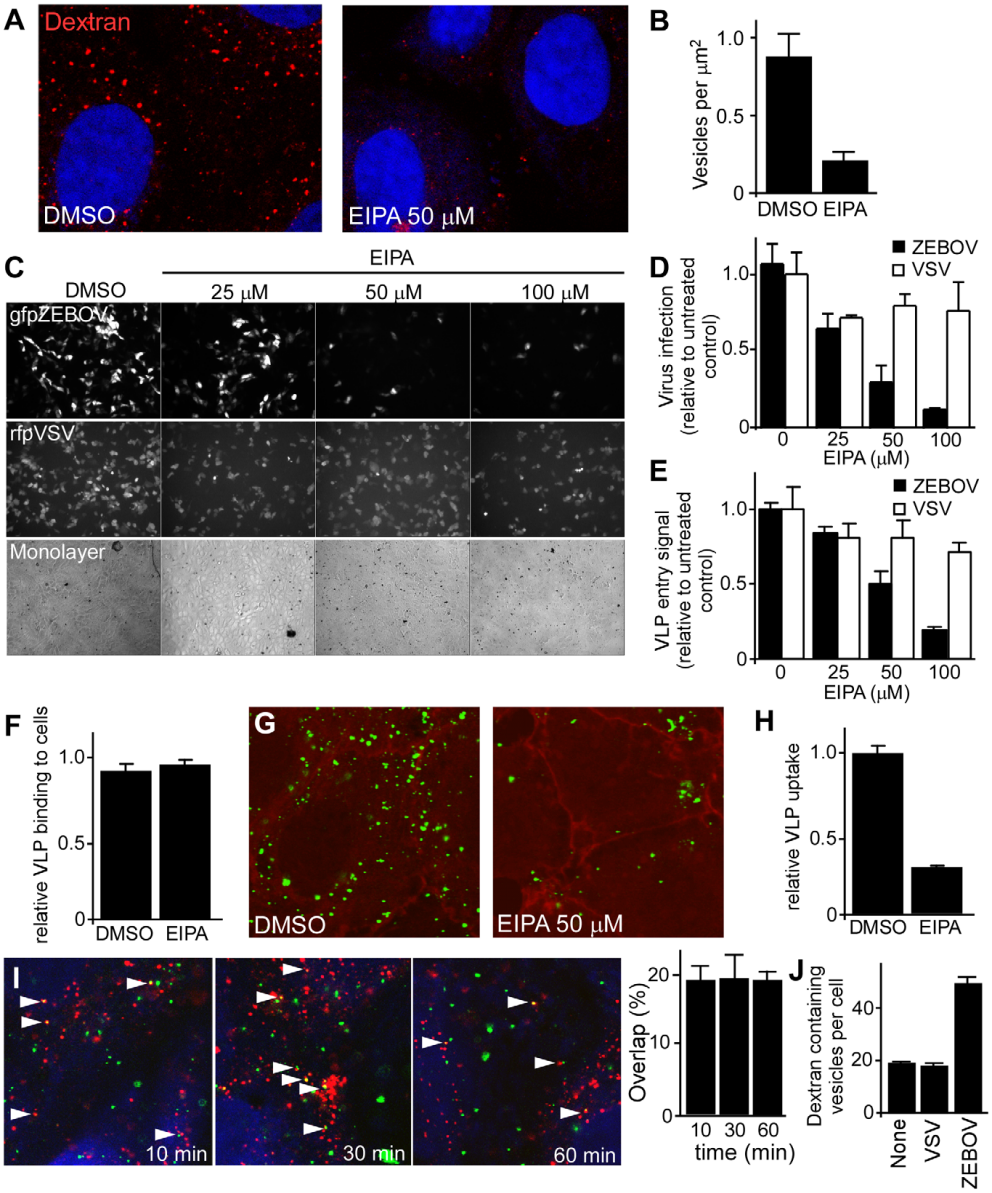
ZEBOV uptake and infection is inhibited by EIPA and VLP uptake is associated with dextran containing vesicles. (**A**) **EIPA inhibits dextran accumulation into vesicles.** Vero cells were treated with DMSO or EIPA (50 uM) for 30 min. Subsequently, cells were incubated with Alexafluor_594_-labeled dextran (1 mg/ml) in the presence of the inhibitor. After 30 min, cells were washed, fixed and observed by confocal microscopy. Nuclei (blue) were stained with DAPI. Images were taken using a 100× oil immersion objective lens. (**B**) Accumulation of dextran in cells was analyzed by counting the total number of macropinocytic vesicles (occupying >0.25 µm^2^ in images) relative to the area occupied by the cell. (**C**) **EIPA blocks ZEBOV infection.** Vero cells were pre-treated with the indicated concentrations of EIPA, followed by incubation with gfpZEBOV (top panel) or rfpVSV (middle panel) each at MOI of 0.1 in the continued presence of the drug. Control cells received DMSO instead of the drug. After 24 h, cells were washed and fixed. Virus infection was determined by counting fluorescent foci. Cell monolayer integrity was confirmed by phase-contrast microscopy (bottom panel). (**D**) Quantitation of data shown in (C). Solid bars represent gfpZEBOV and open bars represent rfpVSV. Data were normalized to the average number of foci seen for untreated cells. Similar results were obtained when HEK293T cells were used (not shown). (**E**) **EIPA-mediated block is at the entry step of infection.** The mechanism of the EIPA-mediated inhibition of infection was examined by performing entry assays. HEK293T cells were pre-treated with the indicated concentrations of EIPA for 1 h followed by incubation with ZEBO-VLP (solid bars) or VSV-VLP (open bars) for an additional 3 h in the continued presence of the drug. Subsequently, cells were washed, and luciferase activity was measured for each sample. The results are expressed as luciferase activity relative to that in control (DMSO-treated) cells. The data represents average ± st.dev. of 3 independent experiments each performed in duplicate. (**F**) **EIPA does not affect ZEBO-VLP binding to cells.** HEK293 cells were pre-treated with EIPA (50 µM) for 1 h at 37°C, followed by incubation with ZEBO-VLPs for 10 min at room temperature. Cells were then washed to remove unbound virus, resuspended in luciferase assay buffer containing triton X-100 detergent, and luciferase activity was measured. Data were normalized to luciferase activity in vehicle-treated samples. Each data point represents mean ± st.dev. of 3 experiments. (**G**) **EIPA treatment inhibits cellular uptake of ZEBO-VLPs.** Vero cells were treated with DMSO or EIPA (50 µM) for 30 min. Subsequently, cells were incubated with gfpZEBO-VLP (green) in the presence of the inhibitor. After 30 min, cells were washed, fixed and the cell periphery was visualized by staining with phalloidin (red), staining cortical actin. Images were taken by confocal microscopy using 100× oil immersion objective lens. Only the mid-optical section representing the cell interior is shown. (**H**) VLP uptake was quantified by counting the total number of internalized VLPs in cells in each image (4–6 cells/image). A total of 10 images were analyzed for each sample. The data are presented as average number of VLPs per cell ± st.dev. (**I**) **Internalized ZEBO-VLPs colocalize with dextran.** HEK293T cells were incubated with gfpZEBO-VLP at 16°C. After 15 min, samples were washed and incubated with Alexafluor_594_-labeled dextran 10,000 W (1 mg/ml) at 37°C. At indicated time intervals, cells were fixed and analyzed by confocal microscopy. Each image represents a mid optical section. Arrowheads indicate examples of association between gfpZEBO-VLPs (green) with dextran-containing vesicles (red). Nuclei (blue) were stained with DAPI. **Quantitation of VLP colocalization** (right panel). Multiple sections of each image were analyzed for VLPs that exhibited colocalization with dextran and their number expressed as percent of total VLPs in those sections. At least 10 images (5–6 cells/image) were analyzed for each sample. Mean ± st.dev. are shown. Note: this is likely an underestimate of the association as VLPs out of the plane or close to but not completely overlapping dextran positive vesicles were not counted. (**J**) **ZEBOV induces dextran uptake by cells.** Vero cells were incubated with replication-competent VSV or ZEBOV (MOI  = 5) for 15 min. Control cells were incubated with growth medium alone (none). Cells were then washed and incubated with medium containing fluorescently-labeled dextran 10,000 MW (1 mg/ml). After 30 min, cells were washed and fixed. Images were then taken by confocal microscopy using a 100× oil immersion objective lens, and the number of dextran-containing vesicles in individual cells were counted. For each sample, at least 10 images (≥25 cells) representing randomly selected fields were analyzed. The data represent mean ± st.dev. of dextran-containing vesicles/cell. A similar outcome was observed when HEK293T cells were incubated with ZEBO-VLP (not shown).

To assess the involvement of macropinocytosis, the effect of EIPA (5-(N-ethyl-N-isopropyl amiloride; Pubchem:1795) on ZEBOV infection was determined. EIPA, an amiloride, is a potent and specific inhibitor of Na^+^/H^+^ exchanger activity important for macropinosome formation [34], [35], [36], [37]. Consistent with this activity, EIPA caused a significant reduction (>80%) in the uptake of high molecular weight dextran, a marker of macropinosomes (Fig. 4A and B). When tested in Vero cells, a dose-dependent inhibition of gfpZEBOV infection was observed in the presence of EIPA (Fig. 4C, top panels; Fig. 4D), while infection by VSV was not significantly affected (Fig. 4C, middle panels; Fig. 4D). As used, EIPA had no significant cytotoxic effect as assessed by cell monolayer integrity (Fig. 4C, bottom panels). Counting of infected cells revealed that VSV infection was inhibited by 30% but increasing the dosage of the drug did not further reduce infection, indicating a small portion of VSV infection may occur through an EIPA-sensitive pathway. In contrast, the majority of ZEBOV infection inhibition was dosage dependent, potent and indicative of inhibition of a single uptake pathway (Fig. 4D). Similar results were observed in HEK293T cells (data not shown).

To rule out the possibility that EIPA blocked ZEBOV infection at a post-entry step, the VLP entry assay was used. Here, EIPA treatment had no significant effect on entry of VSV-VLP (P<0.05) while the level of ZEBO-VLP entry was inhibited similarly to that seen for infectious virus (Fig. 4E). The impact of EIPA on virus binding to cells was also tested. Cells were pretreated with EIPA and then incubated with luciferase-containing ZEBO-VLPs for 10 min on ice. Unbound particles were washed away and then the amount of VLP associated with cells was measured by lysis in non-ionic detergent to release virus-encapsulated luciferase. Compared to DMSO-treated (control) cells, no significant difference was observed in luciferase activity in samples that were treated with EIPA, indicating that ZEBO-VLP binding to cells was unaffected (Fig. 4F). Finally, to directly visualize the effect of EIPA on virus uptake, Vero cells treated with DMSO or EIPA were incubated with gfpZEBOV-VLPs. Confocal microscopy revealed that there was a marked drop (3.5-fold) in gfpZEBO-VLP uptake in cells treated with EIPA as compared to that in DMSO-treated cells (Figs. 4G and H).

### ZEBOV induces fluid phase uptake and colocalizes with internalized dextran

Vaccinia virus was shown to induce fluid phase uptake and exhibit colocalization with fluid phase markers such as dextran [36]. To see if a similar induction of fluid phase uptake was seen with ZEBOV, dextran and virus were incubated together on cells and examined by confocal microscopy. Starting within 10 min and continuing until at least 60 min post-binding, at any one time, approximately 20% of internalized VLPs overlapped with dextran (Fig. 4I). An additional 20–30% of the remaining VLPs were also found juxtaposed to vesicles containing dextran, indicating a close association with this compartment. Furthermore, while performing these experiments we observed that cells incubated with ZEBO-VLPs appeared to have more dextran containing vesicles than intact cells. Indeed, when studied in detail, a (2–3 fold) increase in the number of dextran-containing vesicles per cell was seen after incubation with ZEBOV as compared to cells incubated with VSV or medium alone (Fig. 4J). A similar outcome was seen for cells incubated with VLPs (not shown).

### Activity of p53-activated kinase 1 (Pak1) is required for ZEBOV entry

**Figure 5:**
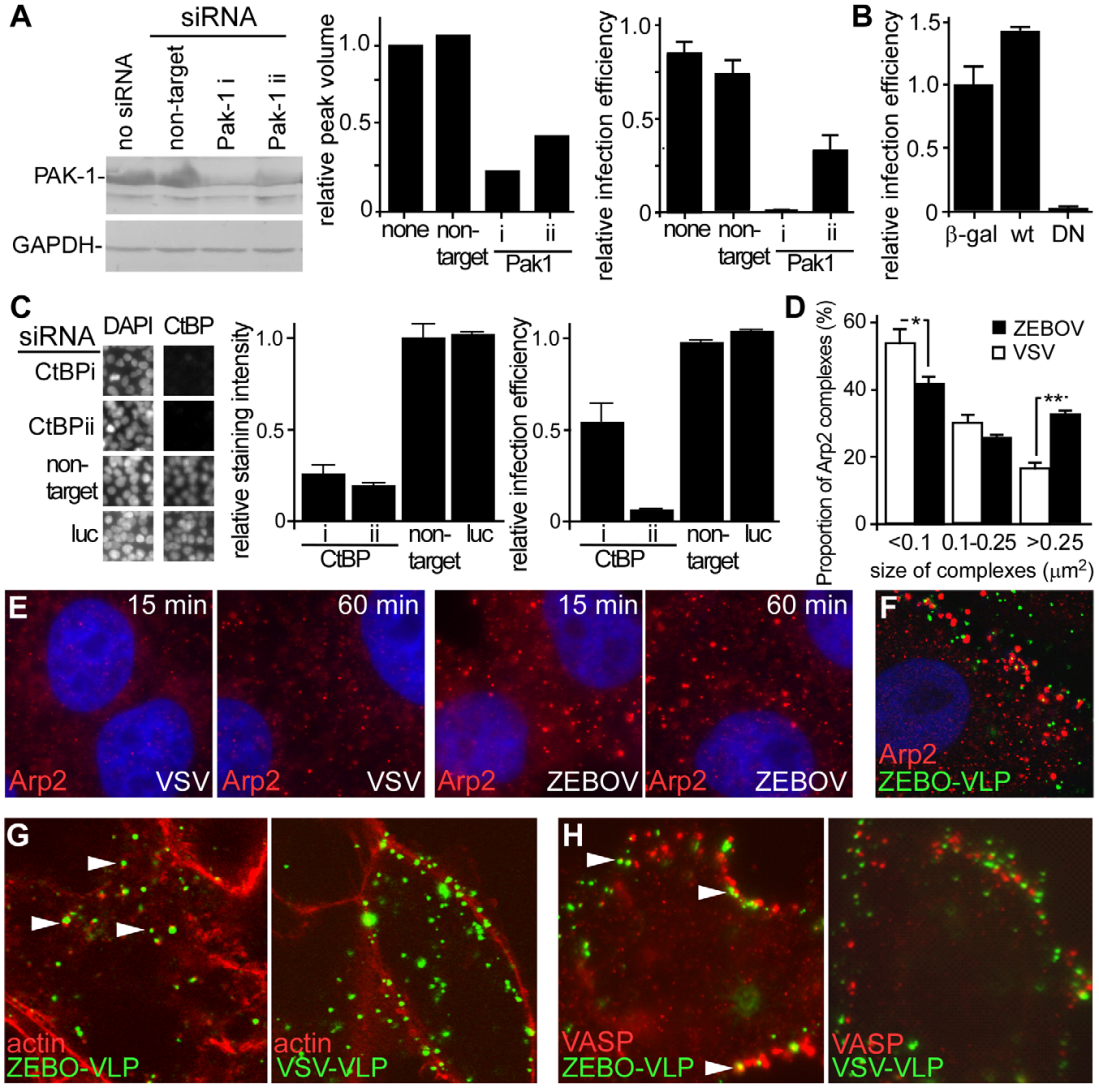
Actin and actin regulatory proteins are important for ZEBOV infection. (**A**) **Suppression of Pak1 by siRNA blocks ZEBOV infection.** HEK293 cells were transfected with siRNA targeting Pak1 (two distinct siRNA, i and ii, used) or non-targeting siRNA. Expression of Pak1 was evaluated by Western blot using an appropriate antibody (Cell Signaling Technology, MA) and relative peak intensity determined by densitometry using a Typhoon scanner and associated software (GE Biosciences, NJ). The impact of Pak1 suppression on gfpZEBOV infection was then determined and expressed relative to untransfected controls. (**B**) **DN Pak1 reduces ZEBOV infection.** HEK293T cells were transfected with plasmids encoding β-galactosidase (β-gal) or myc/GST-tagged forms of wt Pak1 or DN-Pak1. 36 h later, cells were infected with gfpZEBOV and after 24 h were fixed and stained for myc or GST tags using appropriate primary and secondary antibodies. Cells were then imaged and analyzed as in the methods. The proportion of cells that were expressing each tagged protein and infected by ZEBOV was calculated as a fraction of the total cell population and expressed relative to the infection seen for cells transfected with plasmid encoding β-galactosidase. (**C**) **Suppression of CtBP/BARS by siRNA blocks ZEBOV infection.** HEK293 cells were transfected with siRNA targeting CtBP/BARS (two used, i and ii) or non-targeting or firefly luciferase (luc) targeting siRNA. Expression levels were determined by evaluating immunofluorescent staining intensity of CtBP/BARS in nuclei of each cell (CtBP/BARS is predominantly localized to cell nucleus) and normalizing to the nuclear stain, DAPI and untransfected controls. The left panel shows portion of microscope image with cell nuclei stained with DAPI or CtBP/BARS antibody and center panel shows quantitation of staining from 20,000 cells. Right panel shows impact on infection by ZEBOV-GFP. (**D**) **ZEBOV induces Arp2-nucleation.** Vero cells were incubated in medium without virus or replication-competent infectious ZEBOV (MOI  = 5) for the indicated time. Subsequently cells were washed, fixed, permeabilized and stained for Arp2 protein using a specific antibody. The number and apparent size of Arp2 complexes was analyzed using the Analyze particles function of ImageJ software (http://rsbweb.nih.gov/ij/). While total number of Arp2 clusters did not change, the size distribution was altered by ZEBOV incubation with cells. This was expressed as the number of Arp2 complexes of the size ranges indicated (area occupied in image) relative to the total number of complexes (*-P<0.05, **-P<0.01). (**E**) Images showing Arp2 nucleation. Arp2 (red), DAPI stained nuclei (blue). Images were taken by confocal microscopy using a 100× oil immersion objective lens. (**F**) **ZEBO-VLPs associate with Arp2 complexes.** Vero cells were incubated with gfpZEBO-VLPs (green) for 30 min and then fixed, permeabilized and stained for Arp2 (red) using appropriate antibodies. (**G**) **ZEBO-VLPs associate with actin foci and** (**H**) **VASP protein during cell entry.** Vero cells were incubated with fluorescently-labeled ZEBO-VLPs or VSV-VLPs (green). After 30 min, cells were washed, fixed and permeabilized. For actin staining, cells were incubated with medium containing fluorescently-labeled phalloidin (red). For VASP staining, cells were incubated with anti-phospho-VASP antibody, followed by fluorescently-labeled secondary antibody (red). Arrowheads indicate representative examples of VLP colocalization with actin or VASP. All Images were taken by confocal microscopy using a 100× objective lens.

The above data indicated that cellular uptake of ZEBOV primarily occurs through a pathway that has characteristics of macropinocytosis. Another hallmark of macropinocytosis is its dependence on the activity of Pak1 [9]. Therefore, the role of Pak1 in entry of ZEBOV was investigated. First, we measured the effect of siRNA-mediated suppression of endogenous Pak1 and found that the infection of gfpZEBOV was significantly reduced in cells transfected with Pak1 siRNA (Fig. 5A). To confirm, cells transfected with plasmid encoding wild-type Pak1 or DN Pak1 were challenged with gfpZEBOV. The infection of gfpZEBOV was reduced >95% in cells expressing the DN Pak1 protein than in cells expressing the wild-type Pak1 protein (Fig. 5B). A similar effect of DN Pak1 was observed when ZEBO-VLPs were tested in the entry assay (not shown). The protein CtBP/BARS, is also known as important for macropinocytosis [15], [16] and substitutes for dynamin in promoting vesicle scission from the plasma membrane. Again, siRNA were used to suppress expression and the impact on infection measured. Two independent siRNA were able to suppress expression of CtBP/BARS by >70% and 80% respectively (Fig. 5C, left panels). Infection was also reduced by 50% and 90% respectively (Fig 5C, right). This observation indicated that the suppression of CtBP/BARS expression must cross a threshold before becoming limiting to ZEBOV infection but plays an important role. Both sets of data support roles for Pak1 and CtBP/BARS in ZEBOV infection.

### Actin and actin regulatory factors play a role in ZEBOV entry

Macropinocytosis is heavily actin-dependent. Actin is required for the formation of plasma membrane ruffles in macropinosome formation, as well as trafficking of macropinosomes into the cell [38]. Ligands that utilize macropinocytosis often promote changes in the cell actin dynamics by regulating various cellular proteins involved in controlling F-actin assembly and disassembly. Arp2 protein is an integral component of a multi-protein complex that serves as a nucleation site for *de novo* actin assembly. We observed a significant increase in the size of Arp2-containing complexes shortly after ZEBOV binding to cells (Fig. 5D, E). Analysis of the data indicated a 2-fold increase in the number of large (>0.25 µm^2^) Arp2-containing complexes (Fig. 5D). A similar outcome was seen with cells incubated with VLPs (not shown) and a significant proportion of VLPs were associated with Arp2 complexes (Fig. 5F). Further support for a role of actin in ZEBOV entry came from the observation that gfpZEBO-VLPs were associated with F-actin foci within the interior of the cell but this was not seen for VSV-VLPs (Fig. 5G). Similarly, the gfpZEBO-VLPs were also seen associated with vasodilator-stimulated phosphoprotein (VASP), an actin-associated protein that promotes actin nucleation (Fig. 5H). In each of these cases, VLPs and staining for each marker often did not completely overlap. Instead VLP and actin or VASP often were closely juxtaposed and is consistent with nucleation occurring around vesicles containing the VLP. These observations suggested that the virus actively promotes actin assembly and associates with actin-based structures to facilitate its uptake and/or trafficking.

### Intracellular trafficking of ZEBOV proceeds through early and late endosomes

**Figure 6:**
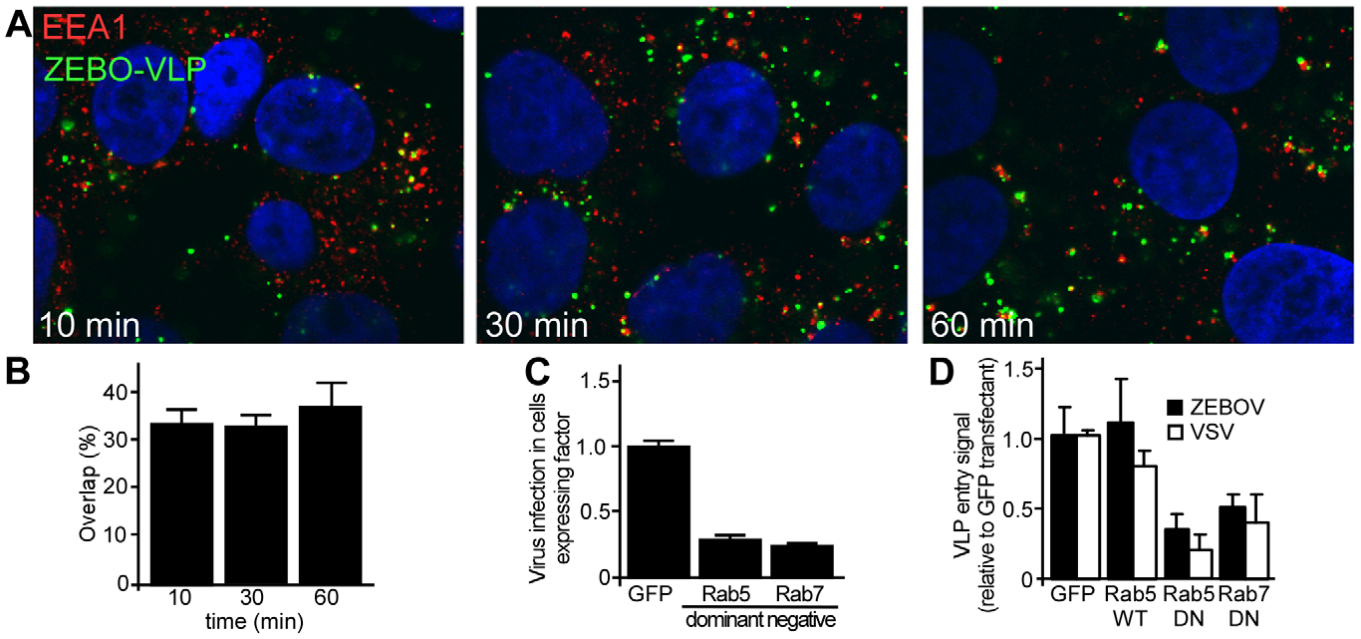
ZEBOV trafficking involves early and late endosomes. (**A**) **ZEBO-VLPs colocalize with vesicles bearing early endosomal antigen-1 (EEA1) shortly after internalization.** HEK293T cells were incubated with fluorescently-labeled ZEBO-VLPs (green) for 10 min at 16°C. After washing to remove unbound VLPs, fresh growth medium was added to cells, which were then incubated at 37°C. At the indicated time cells were fixed, permeabilized and stained for EEA1 (red). Nuclei (blue) were stained with DAPI. Images were taken by confocal microscopy using a 100× oil immersion objective lens. A representative image of mid-optical z-section is shown for each time point. (**B**) **Quantitation of VLP colocalization.** VLPs colocalized with EEA1 were counted and expressed as percent of total VLPs in image sections. At least 10 images (5–6 cells/image) were analyzed for each sample. Mean ± st.dev. are presented in the data. (**C**) **ZEBOV requires Rab5 and Rab7 function.** HEK293T cells were made to express GFP or GFP-tagged forms of DN Rab5, DN Rab7 by plasmid transfection. Twenty-four h post-transfection cells were incubated with wild-type ZEBOV for 48 h. Cells were fixed after 36 h and immunostained for ZEBOV VP40 matrix protein as a marker of infection. Nuclei were stained with DAPI and images were taken by fluorescence microscopy. Image analysis was performed using Cell Profiler software (Broad Inst. MA) as described in methods. The proportion of cells that were expressing each GFP-tagged fusion protein and infected by ZEBOV was calculated as a fraction of the total cell population and averaged for all replicates (>5). Data were normalized to that seen in cells transfected with GFP alone. (**D**) **Rab5 and Rab7 function is necessary for the cell entry step of infection.** To determine the step of infection that was affected by each DN protein, entry assays were performed using HEK293Tcells expressing GFP, or GFP-tagged forms of wild-type Rab5, DN Rab5 or DN Rab7. Cells were incubated with VSV-VLP (open bars) or ZEBO-VLP (solid bars) for 3 h. Subsequently, luciferase activity was measured in each sample and expressed relative to that in control (untransfected) cells. The data represents average ± st.dev. of 3 independent experiments, each performed in duplicate.

The above findings indicated that ZEBOV is primarily internalized by a macropinocytosis-like pathway in Vero and HEK293 cells. However, the subsequent trafficking route to the site of penetration into the cytoplasm remained unknown. We found that fluorescently-labeled ZEBOV particles significantly co-localize with early endosomal antigen-1 (EEA1; OMIM:605070) shortly after incubation with cells (Fig. 6A). At any time up to 60 min after the start of incubation, more than 30% of VLPs were associated with this marker (Fig. 6B). This confirmed a role for endocytic uptake into cells and suggested that following internalization, ZEBOV is delivered to an EEA1-positive compartment, likely sorting endosomes. Typically, the cargo from EEA1-positive compartments is delivered to early endosomes followed by trafficking to late endosomes. These vesicles are characterized by the presence of Rab5 (OMIM:179512) and Rab7 (OMIM:602298) GTPases on the cytoplasmic face of the vesicle, respectively, which play a key role in regulating their trafficking. Consistent with a role for early and late endosomes and in contrast to the lack of effect of DN Eps15 and Cav-1 expression, GFP-tagged DN Rab5 or DN-Rab7 resulted in significant reduction (P<0.001 for each) in infection by gfpZEBOV (Fig. 6C). To determine if the effect was due inhibition of virus entry, VLP entry assays were performed. As compared to the negative control (GFP alone), wild-type Rab5 had no significant effect on entry of either ZEBO-VLP or VSV-VLPs, while there was >50% reduction in entry of both ZEBO-VLPs in cells transfected with either DN-Rab5 or DN-Rab7. The level of entry inhibition seen for ZEBO-VLP was similar to that of VSV-VLPs (Fig. 6C) and indicated that like VSV, ZEBOV is taken up by Rab5-dependent early and Rab7-dependent late endosomes. However, this does not mean that both virus types are present in the same vesicle population but that similar trafficking proteins are required at this stage of endocytosis. Currently, it is unknown whether ZEBOV envelope fusion occurs in late endosomes or further trafficking to a different compartment is needed.

## Discussion

Endocytosis offers an efficient way for viruses to cross the significant physical barrier imposed by the plasma membrane and to traverse the underlying cortical matrix. Viruses have also evolved to target distinct endocytic pathways that are capable of delivering the capsid into the cell cytoplasm at sites suitable to initiate replication and to avoid destructive compartments like the lysosome. Understanding the pathway of virus entry and deciphering the mechanism regulating it is important for understanding viral pathogenesis as virus entry into host cell is the first critical step in pathogenesis of infection.

While there is ample evidence that ZEBOV enters cells through endocytosis in a pH-dependent manner [6], [7], [20], the specific endocytic and trafficking pathways have not been clearly defined. Previous studies to elucidate the ZEBOV entry pathway have produced conflicting findings. Most of these studies relied on the use of retrovirus-based pseudotypes in which the Ebola virus GP is coated onto the surface of a retrovirus capsid containing a recombinant genome. The use of this system overcomes the need for high bio-containment but suffers from not having native virus morphology, GP density, and other biochemical characteristics. One early study on ZEBOV uptake using pseudotyped virus indicated caveolae as important [22] but later work indicated that caveolin activity was not required [39]. In contrast, a recent study concluded that clathrin-mediated endocytosis was the major entry pathway for ZEBOV [21]. While multiple approaches were used, including dominant-negative mutant expression and siRNA to specifically disrupt clathrin-mediated endocytosis, the key data was obtained using lentivirus-based retroviral pseudotypes. In comparison, previous work using wild type virus [20] implicated both clathrin and caveolar endocytosis in entry of ZEBOV. However, only pharmacological inhibitors were used in this study and drug specificity was not examined, making interpretation difficult. Indeed, the only evidence of clathrin involvement in infection was provided using chlorpromazine. Chlorpromazine is a useful drug and there is ample evidence indicating that it disrupts clathrin-coated pits, but it has recently been demonstrated to also interfere with biogenesis of large intracellular vesicles such as phagosomes and macropinosomes [24].

Here, by combining distinct and independent approaches we have performed a detailed analysis of each major endocytic pathway and have obtained, a clear and accurate picture of how ZEBOV enters the cell and identified important cellular proteins that are required. Careful assessment of specificity and functionality of each pathway was performed and correlated to infection and virus uptake. Replication-competent infectious ZEBOV, as well as ZEBO-VLPs (which are morphologically similar to infectious ZEBOV and contain the native matrix protein in addition to GP) were used to study the virus entry mechanism. Drugs were used to inhibit pathways but issues of specificity and pleiotropy were assessed by testing the function of each pathway after treatment. This was done by using independent markers such as transferrin, CTxB and high molecular weight dextran for CME, CavME and macropinocytic uptake respectively. We also assessed the association of fluorescent VLPs with each marker as well as markers of each endocytic compartment being examined. Furthermore, highly specific dominant-negative mutants and/or siRNAs were also used to corroborate the data obtained by pharmacological inhibitors. Importantly, throughout this work a sensitive contents-mixing virus entry assay was used in discriminating against blocks in virus entry versus blocks in downstream steps in the infection cycle. This is particularly important to do when using drugs that often affect multiple cellular functions. It is noteworthy that in each case, virus infection with wild type or the GFP-expressing ZEBOV correlated exactly with the outcomes of the VLP-based assays. This approach gives a highly detailed view of the mechanism of ZEBOV uptake into cells.

Unlike previous studies [20], [21], [22], we found no evidence for the involvement of either CME or CavME in ZEBOV entry and infection. However, there was strong association of fluorescently-labeled ZEBO-VLPs with lipid rafts, and a marked reduction of ZEBOV infection by MBCD or nystatin, as reported previously [32]. This signified that cholesterol-rich lipid raft domains are required for productive entry of the virus. However, cholesterol-rich membrane microdomains play important roles in many forms of endocytosis including caveolae-dependent, non-clathrin/non-caveolar pathways, and macropinocytosis [38], [40]. Our previous work indicated that entry of ZEBOV was dependent on signaling through PI3K and Rac1 [25], which are important regulators of macropinocytosis [38]. Work by others also showed that Rho GTPases play a role in ZEBOV uptake [23]. Each of these cellular signaling proteins are known to be important in macropinocytosis. Macropinocytosis is also distinguished from the other pathways principally by criteria that include actin-dependent structural changes in the plasma membrane, regulation by PI3K, PKC, Rho family GTPases [9], [13], [33], Na^+^/H^+^ exchangers, Pak1, actin, actin regulatory factors, involvement of CtBP/BARS [14], [38] as well as ligand-induced upregulation of fluid phase uptake and colocalization of the internalized ligand with fluid phase markers [14], [36], [37]. In our examination of ZEBOV entry mechanism, we found that EIPA, a potent and specific inhibitor of the Na^+^/H^+^ exchanger [34], [35], [36], [37] blocked ZEBOV infection and entry. Furthermore, ZEBOV caused significant induction of dextran uptake (a fluid phase marker) and the internalized virus particles colocalized with dextran. Pak1 regulates macropinocytosis by promoting actin remodeling and macropinosome closure through phosphorylation of proteins LIMK and CtBP/BARS, respectively [9], [16]. We found that suppression of both Pak1 and CtBP/BARS activity by siRNA or expression of a DN form of Pak1 reduced virus entry and infection.

Actin plays a central role in formation and trafficking of macropinosomes. Actin remodeling is a key event during macropinocytosis and is often triggered by stimuli that promote macropinocytosis. Arp2, among other actin regulatory proteins, has been implicated in macropinocytosis [9]. Arp2 also plays an important role in actin remodeling. It is an integral component of a large multi-protein complex that forms in response to stimuli that trigger actin assembly, and serves as a nucleation site for assembly of actin monomers to form F-actin [41]. We observed a significant increase in the size of the Arp2-containing complexes shortly after ZEBOV binding to cells, indicating stimulation of actin nucleation by the virus. The increase in Arp2 nucleation paralleled an increase in large dextran containing vesicles inside cells corresponding to macropinosomes. This activity appears to be associated with the ZEBOV glycoprotein as VLPs were also capable of inducing a similar increase in Arp2 nucleation and dextran uptake (data not shown). Additionally, we found marked association of fluorescently-labeled ZEBO-VLPs with F-actin foci, as well as with the Arp2-containing complexes and actin-regulatory protein, VASP, that resides in membrane ruffles and promotes actin foci formation. Together, these data provide evidence for a role of actin in ZEBOV entry and suggest that the virus can actively promote localized actin remodeling to facilitate its uptake through macropinocytosis or a similar mechanism.

Despite using multiple approaches, we found no evidence for a role of dynamin in ZEBOV entry. Dynamin is a large GTPase that is involved in scission of newly-formed endocytic vesicles at the plasma membrane [42], [43]. Dynamin-independent entry of ZEBOV further ruled out roles for clathrin or caveolae-mediated pathways as both require dynamin activity [38], [44], [45]. In contrast, the majority of studies suggest that macropinocytosis is independent of dynamin [9]. Recently a novel mechanism has been described for scission of shigatoxin-containing vesicles in which Arp2-dependent actin-triggered membrane reorganization directly leads to vesicle severance [46]. As indicated above, we observed a marked increase in the size of Arp2 complexes shortly after incubation with ZEBOV and a significant association of ZEBO-VLPs with these and F-actin foci but it is unclear if this resulted in membrane scission. In addition, several reports have indicated that C-terminal binding protein (CtBP/BARS), originally identified as a nuclear transcription factor, likely replaces dynamin in scission of nascent macropinosome from the plasma membrane [15], [16]. As discussed above, suppression of CtBP/BARS by siRNA reduced infection and is consistent with the requirement for macropinocytosis in ZEBOV infection.

Interestingly, ZEBOV VP40-based VLPs bearing VSV envelope glycoprotein were found to enter cells through clathrin-mediated endocytosis, as has been reported for the wild-type virus [27]. This suggested that the choice of the internalization pathway is primarily determined by envelope glycoprotein specificity. This is in contrast to a study in influenza virus, where profound differences were seen in the entry characteristics of early passage filamentous virus compared to the laboratory grown spherical isolates that tend to use clathrin-mediated endocytosis [47]. These data indicated a more pronounced role of virion morphology on the choice of endocytic pathway. The apparent reason for this discrepancy is not clear but may relate to the differences in biological characteristics of the viruses and/or cell types used in the two studies.

Overall, our data provide strong evidence that in HEK293T and Vero cells infection by ZEBOV occurs by a process that is closely related to macropinocytosis. We cannot say that entry occurs exclusively by this pathway, but that its disruption blocks the majority of infection and particle uptake. Our work also indicates that clathrin and/or caveolar endocytosis play at most, only a minor role in infection by wild type virus.

A few other viruses as well as bacteria, require macropinocytosis to establish infection [14]. Each uses different mechanisms to induce macropinocytosis. Vaccinia virus has been shown to trigger macropinocytosis by mimicking apoptotic bodies [36]. In contrast, Coxsackie virus and adenovirus activate macropinocytosis by binding to the cell surface proteins occludin and integrin αV, respectively [48], [49]. The mechanism by which ZEBOV triggers macropinocytosis is currently unknown but likely involves GP interaction with cell receptors. Axl (a receptor tyrosine kinase) and integrin βI have been suggested to act as virus receptors [50], [51]. Although, the role of Axl or integrin βI has not been studied in the context of macropinocytosis, there is evidence that several other receptor tyrosine kinases and integrins can trigger macropinocytosis [52], [53], [54]. Therefore, it will be important to analyze the role of Axl and/or integrin βI in this context.

After formation, macropinosomes traffic further into the cytoplasm and may acquire new markers and/or undergo heterotypic fusion with other vesicles of the classical endolysosomal pathway thereby successively transferring the cargo to more acidic compartments such as early and late endosomes [38], [55]. Consistent with this, we found that ZEBO-VLPs co-localized with EEA1-positive vesicles soon after binding [25]. Interestingly, the timing of colocalization of VLPs with EEA1 positive vesicles coincided with their appearance in dextran-containing macropinosomes (within 10 min after binding). Possible explanations may be that the macropinosomes acquire EEA1 shortly after formation or that they undergo prompt fusion with EEA1 positive vesicles. Our data also provided evidence that ZEBOV infection and entry was dependent on Rab5 and Rab7 function, indicating the involvement of early as well as late endosomes in ZEBOV uptake and infection. While a role of early endosomes in Ebola virus entry has not been previously reported, our finding that ZEBOV is trafficked to late endosomes is consistent with prior studies that showed inhibition of Ebola pseudovirion infection by dominant-negative Rab7 [56] and proteolytic processing of Ebola GP1 by late endosome-resident cathepsins [6], [7]. However, it is important to note that many distinct endocytic vesicles associate with Rab5 and Rab7 during maturation but differ by the ligands they carry [57]. This explains why transferrin, a marker of CME, was never seen associated with ZEBOV containing vesicles, even though both require Rab5 and Rab7 for endocytosis.

The intracellular trafficking of the macropinosome is not well understood and existing data provide evidence both for and against the involvement of classical endolysosomal pathway [58]. However, little mechanistic information is available with respect to virus entry by macropinocytosis. Prior to our work only one study analyzed trafficking in any detail, using vaccinia virus and found that virus particles did not colocalize with markers of classical endolysosomal pathway [59]. This difference is likely due to the fact that ZEBOV requires transport to an acidic compartment for membrane fusion while vaccinia virus, which is a pH-independent virus, may undergo nucleocapsid release prior to fusion of macropinosomes with more acidic compartments of the endolysosomal pathway. Our findings now add novel and valuable information regarding macropinosome trafficking mechanism in general and in the context of virus entry.

In conclusion, the evidence presented here demonstrates that ZEBOV utilizes a macropinocytosis-like pathway as the primary means of entry into HEK293T and Vero cells. Once taken up by endocytosis, virus trafficking occurs through early and then late endosomes; however, the exact site where envelope fusion and nucleocapsid release occur is unknown. We do not know if ZEBOV and other filoviruses follow the same pathway into other cell types, like macrophages, that are thought to be a primary target for infection. However, most cell types are capable of macropinocytosis and it is likely that the same or a similar pathway will be used. These findings are important as they not only provided a detailed understanding of ZEBOV entry mechanism, but also identified novel cellular factors that may provide new potential targets for therapies against this virus. It will be important to determine if other filoviruses share the same pathway. If so, it may be possible to develop broad-spectrum therapies that temporarily block this pathway in cells.

## Materials and Methods

### Cells and culture

Human Embryonic Kidney HEK293T and Vero cells were maintained in Dulbecco's modified Eagle's (DMEM) medium supplemented with 10% fetal bovine serum (Gemini Bioproducts, GA), 1% non-essential amino acids (Sigma, MO) and 1% penicillin-streptomycin solution (Sigma, MO).

### Reagents and antibodies

All pharmacological inhibitors were purchased from Calbiochem (San Diego, CA) or Sigma (St. Louis, MO). Stock solutions were prepared either in water, DMSO, or methanol, as per manufacturer's recommendation, and stored at −80°C in small aliquots. Alexafluor-labeled reagents including cholera toxin B subunit, transferrin, dextran (10,000 MW) and secondary antibodies were from Invitrogen (Eugene, OR). Specific antibodies against clathrin light chain, caveolin, dynamin, cholera toxin B, Arp2, CtBP/BARS, phospho-VASP and Pak1 were purchased from Santa Cruz Biotechnology, Inc. (Santa Cruz, CA) or Cell Signaling Technology (Beverly, MA). siRNA were from Qiagen (Valencia, CA) and transfections were performed using Dharmafect transfection reagent 1 according to the manufacturer's (Dharmacon, Lafayette, CO) instructions. 4–6 pmol of siRNA were used per transfection of cells in 0.1 ml of medium per well of a 96-well plate. Assays were performed 48 h after transfection.

### Plasmid constructs

All plasmids were prepared using Qiagen kits or by CsCl gradient centrifugation following standard procedures. The plasmid encoding VSV-G envelope glycoprotein (pLP-VSVG) was purchased from Invitrogen. Construction of the plasmid encoding Nef-luciferase fusion protein (pCDNA3-nef-luc) has been described previously [60]. Plasmids encoding ZEBOV matrix protein (VP40), ZEBOV envelope glycoproteins were kindly provided by Christopher Basler (Mount Sinai School of Medicine), Paul Bates (University of Pennsylvania), and Luis Mayorga (Universidad Nacional de Cuyo, Argentina) respectively. Plasmids expressing dominant-negative Eps15, caveolin-1, and dynamin-2 K44A have been described previously [19]. The Pak1 expression plasmids were obtained from Addgene (Cambridge, MA).

### Production of virus-like particles (VLPs) containing Nef-luciferase fusion protein

ZEBOV-VLPs were produced by co-transfecting HEK293T cells with plasmids encoding ZEBOV matrix (VP40) protein, ZEBOV envelope glycoproteins, and Nef-luciferase fusion protein using the calcium phosphate method. For VSV-VLP, plasmid encoding ZEBOV glycoproteins was replaced with one encoding VSV-G. Cell culture supernatant was collected 48 h after transfection and cell debris was cleared by centrifugation (1,200 rpm for 10 min at 4°C). Subsequently, VLPs were purified by centrifugation (25,000 rpm in SW28 rotor for 3.5 h at 4°C) through a 20% (w/v) sucrose cushion in PBS. The VLP pellet was resuspended in 0.01 volume of DMEM, aliquoted and stored at 4°C. Assays were performed within 2–3 days after purification of VLPs.

### VLP entry assay

HEK293T cells were used for contents mixing assays to measure nucleocapsid release into the cell cytoplasm. The cells were removed from plates by trypsin treatment, pelleted by centrifugation and then resuspended in fresh medium. Cells (10^6^ per assay point) were mixed with nef-luciferase containing VLPs in a volume of 0.2 ml and incubated at 37°C on a rotating platform for 3 h. Subsequently, the cells were washed 2–3 times with DMEM to remove the unbound VLPs and the final cell pellet was resuspended in 0.1 ml of luciferase assay buffer lacking detergent (Promega, WI). Luciferase activity was then measured using a Turner Design TD 20/20 luminometer and expressed as counts/sec.

To study drug activity on virus entry, cells were pre-treated with drug for 1 h, followed by incubation with VLPs in the continued presence of the drug. Virus entry was then measured as described above. For measuring the effect of ectopic gene expression, cells were transfected with the control plasmid or one encoding the protein of interest. Cells were then used for entry assays 36 h after transfection. Typical transfection efficiency was 50–70%.

### Cultivation of ZEBOV and determination of virus titer

Wild type ZEBOV (Mayinga strain) was provided by Michael Holbrook (UTMB, TX) and the recombinant virus encoding GFP (gfpZEBOV) was from Heinz Feldman (NIH, Rocky Mountain Laboratory, MT). The virus was cultivated on Vero-E6 cells by infection at an MOI of approximately 0.1. All infected cells expressed GFP approximately 24 h post-infection. Culture supernatants were collected after 7 d and clarified by centrifugation at 2000 x g for 15 min. Virus titer was determined by serial dilution on Vero-E6 cells. Cells were incubated with virus for 1 h and then overlaid with 0.8% tragacanth gum in culture medium. 10 d post-infection cells were fixed with formalin, and stained with crystal violet 10 d post-infection for plaque counting. All experiments with ZEBOV were performed under biosafety level 4 conditions in the Robert E. Shope BSL-4 Laboratory at UTMB.

### Virus infection assays

Cells were pre-treated with inhibitors for 1 h and then incubated with gfpZEBOV at 37°C for 2 h (except in the case of MBCD and nystatin, where cells were washed to remove the inhibitors prior to incubation with the virus). Subsequently, the unbound virus particles were removed by washing with PBS, and cells incubated in fresh growth medium. Twenty-four h later, cells were washed and fixed with 10% formalin for 48 h. Images were taken by epifluorescence microscopy and infected foci counted. Counting was performed using the Cell Profiler software package [61]. The processing pipeline used by the software is available upon request.

### Immunofluorescence staining and microscopy of VLP uptake into cells

HEK293T or Vero-E6 cells were cultivated overnight on chambered coverglass slides (Nunc, Rochester, NY) at a density of 50%. The following day, cells were incubated with GFP-tagged ZEBO-VLPs. Cells were then washed three times in DMEM and fixed in 4% fresh paraformaldehyde in PBS. After one wash in PBS residual paraformaldehyde was neutralized by addition of 0.1 M glycine buffer, pH 7.4 and cells were permeabilized using 0.1% Triton X-100 for 1 min at room temperature. For immunofluorescence, cells were incubated with the appropriate primary antibody, typically diluted 1∶200 in PBS. After washing in PBS, the cells were then incubated with the indicated secondary Alexafluor conjugated secondary antibody. Cells were imaged using a Nikon TE Eclipse inverted microscope with a 100× oil immersion lens or a Zeiss LSM 510 confocal microscope in the UTMB optical imaging core.

### Analysis of the impact of ectopic gene expression on infection by replication competent viruses

Cells were transfected with plasmids encoding GFP-tagged forms of the protein of interest. For work with Pak1, myc-tagged or GST-tagged expression constructs were also used. After 24 h, the cells were challenged with wild type ZEBOV. After an additional 48 h, the cells were fixed in formalin. Cells were then stained for ZEBOV VP40 using a rabbit polyclonal antiserum (Ricardo Carrion, Southwest Foundation for Biomedical Research, San Antonio, TX) followed by an Alexa_633_ secondary antibody. For Pak1 work, cells were also stained for myc-tag or GST-tag using the corresponding primary and a fluorescently labeled secondary antibody. Cell nuclei were also stained using DAPI (Invitrogen). Images were taken using an epifluorescence microscope and the intensity of GFP fluorescence and VP40 staining was evaluated on a cell per cell basis using the Cell profiler software package [61]. For this, cells were first identified by DAPI staining of the cell nuclei. Then cytoplasmic fluorescence intensity for GFP and VP40 staining was determined. The algorithm pipeline used for this part of the analysis is available from R. Davey upon request. The output data, which gives intensities on a scale of 0 to 1, was converted to a scale of 0–1024 using Excel. This data file was then converted to a text file and processed using A2FCS software, which is part of the MFI/FCS Verification Suite (Purdue University) and is available at http://www.cyto.purdue.edu/flowcyt/software/Catalog.htm. This conversion makes the data accessible to conventional FACS analysis software. The data were then analyzed using FlowJo v7.5 (http://www.flowjo.com). Gates were set to exclude cells that were not infected and not expressing the tagged protein, as determined in control experiments. These were done for normal cells infected by virus but not stained, cells not infected by virus but stained with VP40 specific antibody and cells expressing the tagged protein and not infected (stained with antibody against the tagged protein when used). To quantitate the infection dependency of ZEBOV on expression of each construct, the proportion of cells that were expressing each tagged protein construct and infected by ZEBOV was calculated as a fraction of the total cell population. While not used here, this analytical approach can be extended further by setting gates for low, moderate and high levels of ectopic gene expression and then correlating the outcome on infection.

To measure the effect of DN-Cav1 on 10A1 MLV infection, HEK293 cells were transfected with plasmid encoding GFP or GFP tagged DN-Cav1 protein. Thirty six hours after transfection cells were infected with 10A1 MLV pseudotype encoding truncated CD4 receptor (Miltenyi, Germany) as a marker for infection. 36 h after infection the cells were stained for CD4 expression with PE-labeled mouse anti-human CD4 antibody (BD Pharmingen Cat#555347). After 1 h the cells were washed in PBS and fixed in 4% paraformaldehyde. Cells were stained with DAPI to identify cell nuclei and were imaged by a Nikon TE eclipse microscope with an automated motorized stage. To analyze the effect of DN-Cav1 on infection, images were analyzed using Cell Profiler software (Broad Institute, Cambridge, MA) to detect total cells, cells expressing the expression construct and those infected by detection of CD4. Analysis was then performed as above.

## References

[1] Hoenen T, Groseth A, Falzarano D, Feldmann H (2006). Ebola virus: unravelling pathogenesis to combat a deadly disease.. Trends Mol Med.

[2] Leroy EM, Rouquet P, Formenty P, Souquiere S, Kilbourne A (2004). Multiple Ebola virus transmission events and rapid decline of central African wildlife.. Science.

[3] Ellis DS, Simpson IH, Francis DP, Knobloch J, Bowen ET (1978). Ultrastructure of Ebola virus particles in human liver.. J Clin Pathol.

[4] Feldmann H, Klenk HD, Sanchez A (1993). Molecular biology and evolution of filoviruses.. Arch Virol.

[5] Dolnik O, Kolesnikova L, Becker S (2008). Filoviruses: Interactions with the host cell.. Cell Mol Life Sci.

[6] Chandran K, Sullivan NJ, Felbor U, Whelan SP, Cunningham JM (2005). Endosomal proteolysis of the Ebola virus glycoprotein is necessary for infection.. Science.

[7] Schornberg K, Matsuyama S, Kabsch K, Delos S, Bouton A (2006). Role of endosomal cathepsins in entry mediated by the Ebola virus glycoprotein.. J Virol.

[8] Doherty GJ, McMahon HT (2009). Mechanisms of Endocytosis.. Annu Rev Biochem.

[9] Mercer J, Helenius A (2009). Virus entry by macropinocytosis.. Nat Cell Biol.

[10] Pelkmans L, Helenius A (2003). Insider information: what viruses tell us about endocytosis.. Curr Opin Cell Biol.

[11] Kartenbeck J, Stukenbrok H, Helenius A (1989). Endocytosis of simian virus 40 into the endoplasmic reticulum.. J Cell Biol.

[12] Weed SA, Parsons JT (2001). Cortactin: coupling membrane dynamics to cortical actin assembly.. Oncogene.

[13] Falcone S, Cocucci E, Podini P, Kirchhausen T, Clementi E (2006). Macropinocytosis: regulated coordination of endocytic and exocytic membrane traffic events.. J Cell Sci.

[14] Kerr MC, Teasdale RD (2009). Defining macropinocytosis.. Traffic.

[15] Haga Y, Miwa N, Jahangeer S, Okada T, Nakamura S (2009). CtBP1/BARS is an activator of phospholipase D1 necessary for agonist-induced macropinocytosis.. Embo J.

[16] Liberali P, Kakkonen E, Turacchio G, Valente C, Spaar A (2008). The closure of Pak1-dependent macropinosomes requires the phosphorylation of CtBP1/BARS.. Embo J.

[17] Harbison CE, Lyi SM, Weichert WS, Parrish CR (2009). Early steps in cell infection by parvoviruses: host-specific differences in cell receptor binding but similar endosomal trafficking.. J Virol.

[18] Johns HL, Berryman S, Monaghan P, Belsham GJ, Jackson T (2009). A dominant-negative mutant of rab5 inhibits infection of cells by foot-and-mouth disease virus: implications for virus entry.. J Virol.

[19] Kolokoltsov AA, Deniger D, Fleming EH, Roberts NJ, Karpilow JM (2007). Small interfering RNA profiling reveals key role of clathrin-mediated endocytosis and early endosome formation for infection by respiratory syncytial virus.. J Virol.

[20] Sanchez A (2007). Analysis of filovirus entry into vero e6 cells, using inhibitors of endocytosis, endosomal acidification, structural integrity, and cathepsin (B and L) activity.. J Infect Dis.

[21] Bhattacharyya S, Warfield KL, Ruthel G, Bavari S, Aman MJ (2010). Ebola virus uses clathrin-mediated endocytosis as an entry pathway.. Virology.

[22] Empig CJ, Goldsmith MA (2002). Association of the caveola vesicular system with cellular entry by filoviruses.. J Virol.

[23] Quinn K, Brindley MA, Weller ML, Kaludov N, Kondratowicz A (2009). Rho GTPases modulate entry of Ebola virus and vesicular stomatitis virus pseudotyped vectors.. J Virol.

[24] Ivanov AI (2008). Pharmacological inhibition of endocytic pathways: is it specific enough to be useful?. Methods Mol Biol.

[25] Saeed MF, Kolokoltsov AA, Freiberg AN, Holbrook MR, Davey RA (2008). Phosphoinositide-3 kinase-Akt pathway controls cellular entry of Ebola virus.. PLoS Pathog.

[26] Acosta EG, Castilla V, Damonte EB (2009). Alternative infectious entry pathways for dengue virus serotypes into mammalian cells.. Cell Microbiol.

[27] Sun X, Yau VK, Briggs BJ, Whittaker GR (2005). Role of clathrin-mediated endocytosis during vesicular stomatitis virus entry into host cells.. Virology.

[28] Beer C, Andersen DS, Rojek A, Pedersen L (2005). Caveola-dependent endocytic entry of amphotropic murine leukemia virus.. J Virol.

[29] Pang H, Le PU, Nabi IR (2004). Ganglioside GM1 levels are a determinant of the extent of caveolae/raft-dependent endocytosis of cholera toxin to the Golgi apparatus.. J Cell Sci.

[30] Macia E, Ehrlich M, Massol R, Boucrot E, Brunner C (2006). Dynasore, a cell-permeable inhibitor of dynamin.. Dev Cell.

[31] Ebihara H, Theriault S, Neumann G, Alimonti JB, Geisbert JB (2007). In vitro and in vivo characterization of recombinant Ebola viruses expressing enhanced green fluorescent protein.. J Infect Dis.

[32] Bavari S, Bosio CM, Wiegand E, Ruthel G, Will AB (2002). Lipid raft microdomains: a gateway for compartmentalized trafficking of Ebola and Marburg viruses.. J Exp Med.

[33] West MA, Prescott AR, Eskelinen EL, Ridley AJ, Watts C (2000). Rac is required for constitutive macropinocytosis by dendritic cells but does not control its downregulation.. Curr Biol.

[34] Kalin S, Amstutz B, Gastaldelli M, Wolfrum N, Boucke K Macropinocytotic uptake and infection of human epithelial cells with species B2 adenovirus type 35.. J Virol.

[35] Fretz M, Jin J, Conibere R, Penning NA, Al-Taei S (2006). Effects of Na+/H+ exchanger inhibitors on subcellular localisation of endocytic organelles and intracellular dynamics of protein transduction domains HIV-TAT peptide and octaarginine.. J Control Release.

[36] Mercer J, Helenius A (2008). Vaccinia virus uses macropinocytosis and apoptotic mimicry to enter host cells.. Science.

[37] Raghu H, Sharma-Walia N, Veettil MV, Sadagopan S, Chandran B (2009). Kaposi's sarcoma-associated herpesvirus utilizes an actin polymerization-dependent macropinocytic pathway to enter human dermal microvascular endothelial and human umbilical vein endothelial cells.. J Virol.

[38] Mercer J, Schelhaas M, Helenius A. Virus entry by endocytosis.. Annu Rev Biochem.

[39] Simmons G, Rennekamp AJ, Chai N, Vandenberghe LH, Riley JL (2003). Folate receptor alpha and caveolae are not required for Ebola virus glycoprotein-mediated viral infection.. J Virol.

[40] Grimmer S, van Deurs B, Sandvig K (2002). Membrane ruffling and macropinocytosis in A431 cells require cholesterol.. J Cell Sci.

[41] Le Clainche C, Carlier MF (2008). Regulation of actin assembly associated with protrusion and adhesion in cell migration.. Physiol Rev.

[42] Henley JR, Cao H, McNiven MA (1999). Participation of dynamin in the biogenesis of cytoplasmic vesicles.. Faseb J.

[43] Roux A, Uyhazi K, Frost A, De Camilli P (2006). GTP-dependent twisting of dynamin implicates constriction and tension in membrane fission.. Nature.

[44] Mettlen M, Pucadyil T, Ramachandran R, Schmid SL (2009). Dissecting dynamin's role in clathrin-mediated endocytosis.. Biochem Soc Trans.

[45] Nomura R (2005). [Caveolar endocytosis and virus entry].. Uirusu.

[46] Romer W, Pontani LL, Sorre B, Rentero C, Berland L, et al. Actin dynamics drive membrane reorganization and scission in clathrin-independent endocytosis.. Cell.

[47] Sieczkarski SB, Whittaker GR (2005). Characterization of the host cell entry of filamentous influenza virus.. Arch Virol.

[48] Meier O, Boucke K, Hammer SV, Keller S, Stidwill RP (2002). Adenovirus triggers macropinocytosis and endosomal leakage together with its clathrin-mediated uptake.. J Cell Biol.

[49] Coyne CB, Shen L, Turner JR, Bergelson JM (2007). Coxsackievirus entry across epithelial tight junctions requires occludin and the small GTPases Rab34 and Rab5.. Cell Host Microbe.

[50] Shimojima M, Takada A, Ebihara H, Neumann G, Fujioka K (2006). Tyro3 family-mediated cell entry of Ebola and Marburg viruses.. J Virol.

[51] Takada A, Watanabe S, Ito H, Okazaki K, Kida H (2000). Downregulation of beta1 integrins by Ebola virus glycoprotein: implication for virus entry.. Virology.

[52] Li C, Macdonald JI, Hryciw T, Meakin SO (2009). Nerve Growth Factor Activation of the TrkA Receptor Induces Cell Death, by Macropinocytosis, in Medulloblastoma Daoy Cells..

[53] Bryant DM, Kerr MC, Hammond LA, Joseph SR, Mostov KE (2007). EGF induces macropinocytosis and SNX1-modulated recycling of E-cadherin.. J Cell Sci.

[54] Meier O, Greber UF (2004). Adenovirus endocytosis.. J Gene Med.

[55] Hewlett LJ, Prescott AR, Watts C (1994). The coated pit and macropinocytic pathways serve distinct endosome populations.. J Cell Biol.

[56] Meertens L, Bertaux C, Dragic T (2006). Hepatitis C virus entry requires a critical postinternalization step and delivery to early endosomes via clathrin-coated vesicles.. J Virol.

[57] Lakadamyali M, Rust MJ, Zhuang X (2006). Ligands for clathrin-mediated endocytosis are differentially sorted into distinct populations of early endosomes.. Cell.

[58] Jones AT (2007). Macropinocytosis: searching for an endocytic identity and role in the uptake of cell penetrating peptides.. J Cell Mol Med.

[59] Sandgren KJ, Wilkinson J, Miranda-Saksena M, McInerney GM, Byth-Wilson K A differential role for macropinocytosis in mediating entry of the two forms of vaccinia virus into dendritic cells.. PLoS Pathog.

[60] Saeed MF, Kolokoltsov AA, Davey RA (2006). Novel, rapid assay for measuring entry of diverse enveloped viruses, including HIV and rabies.. J Virol Methods.

[61] Carpenter AE, Jones TR, Lamprecht MR, Clarke C, Kang IH (2006). CellProfiler: image analysis software for identifying and quantifying cell phenotypes.. Genome Biol.

